# Carinal Compression from Primary Mediastinal Germ Cell Tumor

**DOI:** 10.7759/cureus.29227

**Published:** 2022-09-16

**Authors:** Kristine Landrian, Kevin R Davidson

**Affiliations:** 1 Department of Internal Medicine, Division of Pulmonary & Critical Care, WakeMed Health & Hospitals, Raleigh, USA

**Keywords:** non-seminomatous germ cell tumor, thoracic anesthesia, airway stenting, interventional pulmonology, mediastinal germ cell tumor

## Abstract

Mediastinal masses can present as a medical emergency when there is central airway obstruction, superior vena cava (SVC) syndrome, direct mediastinal extension of tumor, or obstruction of the central pulmonary vasculature. Diagnostic evaluation may include the need for invasive tissue biopsy under anesthesia, which can pose several distinct risks for patients. Among the many etiologies of mediastinal tumors, primary mediastinal germ cell tumors are a rare form with a favorable prognosis.

## Introduction

Mediastinal masses historically were defined by their anatomic location in either anterior, middle, or posterior mediastinal compartments. The International Thymic Malignancy Interest Group (ITMIG) refined the classification of mediastinal tumor location into prevascular, visceral, and paravertebral compartments with boundaries that are more clearly defined on axial and sagittal CT or MRI imaging [1].

Anatomic designation assists with determining the etiology of a mediastinal mass, although rare causes of mediastinal masses also exist. The location of a mass may also assist with the prediction of future symptoms or risk of airway or vascular compromise with the induction of anesthesia [2]. Depending on location, a biopsy of a mediastinal mass might be performed bronchoscopically, percutaneously, or surgically via mediastinoscopy. Increasing the depth of anesthesia and transition from spontaneous respiration to positive pressure ventilation have several effects on hemodynamics. With the loss of airway reflexes and the induction of positive pressure ventilation with positive end expiratory pressure (PEEP), there is reduced cardiac preload as intrathoracic blood flow is reduced [2,3]. Furthermore, some anesthetic medications have negative inotropic and chronotopic effects on the heart and can lower cardiac output. A mediastinal mass that was causing symptoms, which were partially compensated during spontaneous respiration and without anesthetic can cause imminent cardiovascular collapse during the induction of anesthesia, causes worsening airway obstruction or hemodynamic compromise through these mechanisms [2]. Additionally, other complications may also arise from the presence of a mediastinal mass such as direct cardiac invasion that could lead to arrhythmia or pericardial effusion, cardiac tamponade, compression of the recurrent laryngeal nerve leading to a paralyzed vocal cord, or even paraneoplastic conditions such as syndrome of inappropriate antidiuretic hormone production and neuromuscular weakness from myasthenia gravis [2].

## Case presentation

**Figure 1:**
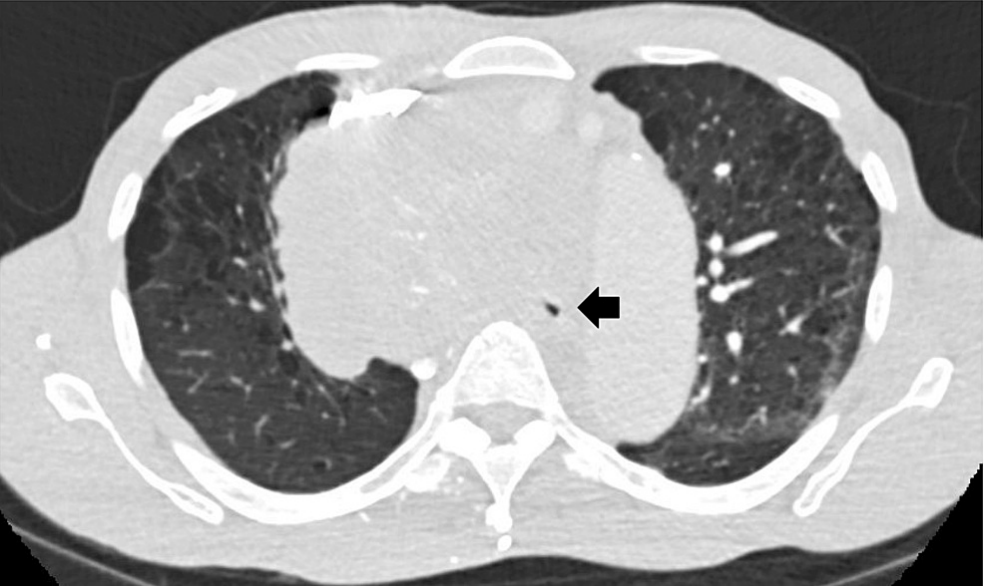
CT scan with large mediastinal mass causing critical compression of the trachea down to 4 mm diameter. The black arrow indicates a critically narrowed tracheal lumen.

**Figure 2:**
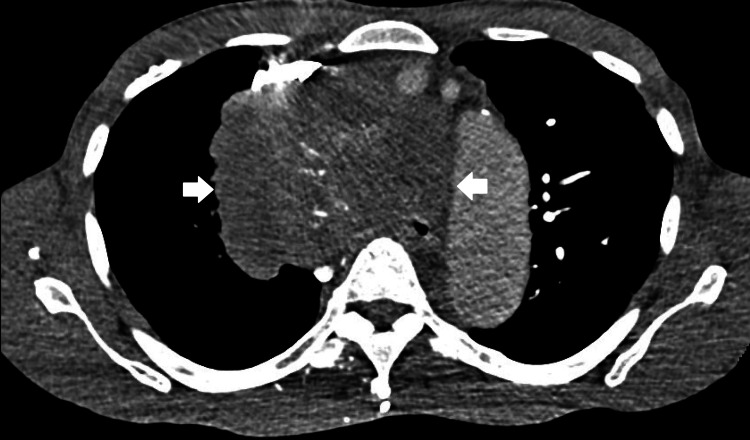
Mediastinal CT scan images. White arrows mark the large mediastinal mass with partial internal calcifications.

**Figure 3:**
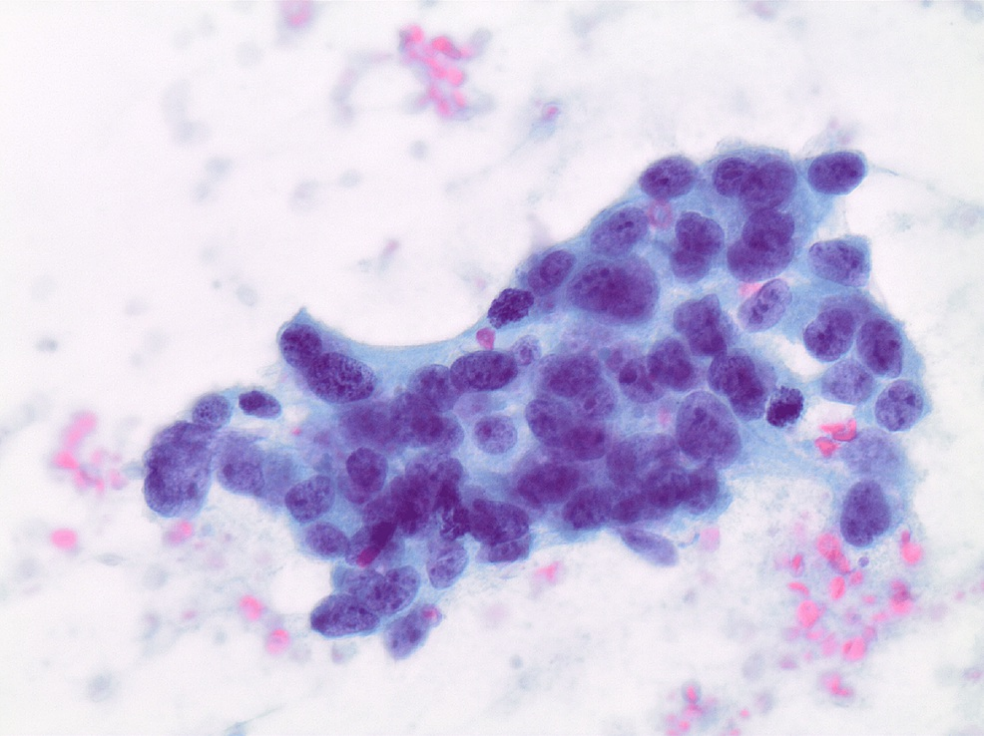
Fine needle aspiration of mediastinal mass with malignant cells, Diff-Quik stain, 60× magnification. Further immunohistochemical testing confirmed non-seminomatous germ cell tumor (NSGCT).

A 64-year-old gentleman with 50 pack-year history of tobacco dependence presented with severe respiratory distress with orthopnea, cough, and right upper extremity swelling. He was hypertensive and hypoxic; blood pressure was 170/111 mmHg, heart rate was 92 beats per minute, and respiration was 30 per minute, and he was afebrile with SpO_2_ of 85% on room air. He had labored breathing and remained in an upright tripod position as his breathing worsened while recumbent. Physical examination revealed bilateral lung wheezes, jugular venous distension, and prominent dilated vessels with arm swelling in bilateral upper extremities consistent with superior vena cava (SVC) syndrome. The patient was able to tolerate a rapid CT scan, which revealed a partially calcified large mediastinal mass measuring 16 cm × 15 cm × 8 cm with critical compression of the distal trachea and carina down to a 4 mm diameter airway along with extrinsic compression of right-sided pulmonary arteries (Figures 1, 2). The patient was placed on non-invasive positive pressure ventilation and brought to the operating room for emergent rigid bronchoscopy and airway stenting. Instead of standard intubation in a semi-upright or supine position, he was positioned at a steep upright seated angle, and additional intravenous (IV) access was established in his lower extremities. With the induction of total intravenous anesthesia, he was intubated directly with a rigid bronchoscope to stent open airways and commenced on jet ventilation. Critical extrinsic compression of the trachea and carina was treated with the deployment of an 18 × 40 mm fully covered self-expanding metal stent within the distal trachea. Conventional transbronchial needle aspiration with 21 g needle of the mediastinal mass revealed non-seminomatous germ cell tumor (NSGCT) (Figure 3). After airway stenting, he was awakened and extubated in the operating room with substantial improvement in dyspnea.

**Figure 4:**
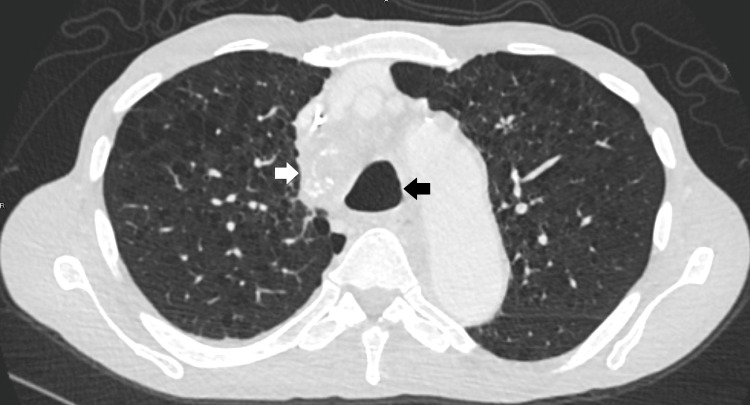
Repeat CT scan after four cycles of chemotherapy and the removal of tracheal stent shows dramatic reduction in mediastinal mass (white arrow), as well as the resolution of tracheal compression (black arrow).

His serum alpha fetoprotein (AFP) was elevated at 1,079 ng/mL (normal <9 ng/mL), and quantitative beta human chorionic gonadotropin (hCG) level was 9 mIU/mL (normal <3 mIU/mL). Testicular examination was normal, and ultrasound of his testes did not reveal any nodules or other abnormalities. He was diagnosed with a primary mediastinal non-seminomatous germ cell tumor (NSGCT) yolk sac subtype, and treated with four cycles of cisplatin, etoposide, and ifosfamide chemotherapy with dramatic reduction in tumor burden. SVC syndrome resolved within initial cycles of chemotherapy, and tracheal stent was removed without difficulty three months later with complete resolution of airway stenosis (Figure 4).

## Discussion

Mediastinal masses can cause symptoms due to airway compression, SVC syndrome, direct mediastinal invasion, pulmonary vasculature obstruction, pericardial effusions, paraneoplastic syndromes, direct mediastinal invasion, and compression of the recurrent laryngeal nerve [1,2]. Patients with airway compression are often misdiagnosed with bronchoconstriction due to asthma or emphysema based on expiratory wheezing from dynamic intrathoracic airway obstruction worsening on exhalation. Therapeutic bronchoscopy with airway stenting may alleviate severe central airway obstruction from compressive masses; however, such cases can pose significant perioperative and anesthetic risks including cardiorespiratory collapse and death. Femoral access extracorporeal membrane oxygenation (ECMO) can be an invaluable tool to increase procedural safety and provide temporary respiratory and circulatory support for patients with central airway obstruction. Our case emphasizes several key procedural and anesthetic challenges to patients presenting with large mediastinal masses including central airway obstruction and SVC syndrome. Care was taken to position him nearly upright and intubate with rigid bronchoscope to reduce the risk of complete airway obstruction after the induction of anesthesia and transition to positive pressure ventilation with loss of airway reflexes [2,3]. Supine positioning alone can increase the amount of hemodynamic compromise or airway obstruction exerted by an anterior or middle mediastinal mass on the pulmonary arteries or central airways [2]. His tripod positioning on presentation suggested that supine positioning would not be well tolerated with loss of airway reflexes on the induction of anesthesia. Although he was able to briefly tolerate laying supine for a CT scan, this was while spontaneously breathing and accompanied by transient worsened dyspnea. A lower extremity IV was placed to ensure rapid medication delivery because of the SVC obstruction, which can delay blood circulation from the upper extremities [2].

The elevated AFP serum markers and imaging were suggestive of a germ cell tumor, which was confirmed on pathology as a yolk sac non-seminomatous germ cell tumor. Primary mediastinal NSGCTs are analogous to non-seminomatous germ cell tumors of the testes but occur in the absence of any testicular pathology. Such tumors are believed to originate from precursor germ cells, which are retained within the primitive ectoderm instead of normal migration into the urogenital ridge during embryogenesis [4]. Another possible hypothesis suggests anomalous reverse migration of germ cell tumors from the gonads into the mediastinum [4]. Although very rare in comparison to lung carcinomas, NSGCT was suggested by the partially calcified tumor within the anterior mediastinum on CT scan, as well as very elevated serum AFP tumor marker. Tumor markers that can be measured in serum to evaluate the possibility of a germ cell tumor include AFP, beta hCG, and lactate dehydrogenase. Germ cell tumors of the mediastinum portend a significantly better prognosis than lung carcinoma as these tumors can often be treated effectively with chemotherapy as shown in this case. The airway stent was effective in palliating his dyspnea until the tumor burden could be reduced with chemotherapy.

## Conclusions

Mediastinal masses can present with symptoms of wheezing, cough, or dyspnea from central airway compression, hoarseness from recurrent laryngeal nerve impingement, SVC syndrome, central vascular obstruction, or direct mediastinal invasion. Successful perioperative management of patients with mediastinal masses requires planning to prevent airway and central vascular compromise with the induction of anesthesia. Primary mediastinal germ cell tumors are rare but carry a much more favorable prognosis and may be suggested by serum tumor marker elevation, as well as calcifications within mediastinal masses on CT scan.
